# Depressive Symptoms and Quality of Life in a Sample of Italian Women with a Diagnosis of Fibromyalgia: The Role of Attachment Styles

**DOI:** 10.1155/2021/5529032

**Published:** 2021-02-16

**Authors:** Cristina Sechi, Loredana Lucarelli, Laura Vismara

**Affiliations:** Department of Pedagogy, Psychology and Philosophy, University of Cagliari, Italy

## Abstract

**Background:**

Women with fibromyalgia (FM) commonly suffer from depression, pervasive fatigue, and pain. The attachment style has been hypothesized to be an important factor for understanding the experience of these symptoms. Therefore, the present cross-sectional study is aimed at investigating the effect of attachment styles in women with a diagnosis of FM on depressive symptoms and quality of life.

**Method:**

Participants were 453 Italian women with a physician's diagnosis of FM with a mean age of 47 years (SD = 10.9). To assess attachment styles, quality of life, and depressive symptoms, women responded, respectively, to the Relationship Questionnaire, the World Health Organization Quality of Life Questionnaire, and the Beck Depression Inventory II.

**Results:**

Our results showed that the incidence of depressive symptoms was elevated, with 59% of women reporting moderate to severe symptoms. Also, the statistical analyses showed that both preoccupied and avoidant/dismissing attachments were related with depression symptoms and low perception of QoL.

**Conclusions:**

Our study demonstrates that, when evaluating the impact of FM on the QoL of women, it is important to consider the complexity of the variables that are at play. Insecure attachment styles and depressive symptoms seem to increase the likelihood of the psycho-social-somatic malaise in FM women.

## 1. Introduction

### 1.1. Background

Fibromyalgia affects about 2.32% of the general population, 90% of whom are women [1]. In Italy, about 2.2% of the general population suffers from FM [2]. FM is characterized by pervasive fatigue and pain, feelings of worries, and difficulties, as well as neurocognitive deficiencies and sleep disorders [3]. FM inevitably influences all aspects of life, including work, quality of life (QoL), and functional ability [4], thereby reducing mental, physical, and social health [5, 6]. QoL may also be impacted by the depressive symptoms that are associated with FM [7].

Indeed, several studies have revealed a life span prevalence of 90% of depressive symptoms and a rate between 62 and 86% of comorbidity with a diagnosis of depression [8]. These percentages significantly differentiate FM patients from both the general population and individuals who suffer from neuropathic pain conditions [9].

Even if it has been demonstrated that more severe symptoms of intensity of depression are associated with lower perceived QoL [10], some scholars have found that variations in QoL are not necessarily aligned to variations in depression and that QoL changes seem to be more gradual than those of depression [11].

Evidence from recent studies suggests that attachment may intervene in these processes [12]. According to the attachment theory [13], several studies investigated how attachment styles may affect mood states and health outcomes [12].

Attachment style is a stable trait throughout adult life; it regulates how people relate to each other and it is related to strategies for managing adverse conditions [13]. According to Bartholomew and Horowitz's model [14], there are four categories of adult attachment style: one attachment style characterized by a positive model of self and other in a relationship (secure) and three insecure styles: one characterized by a negative model of the self and the other (fearful), one characterized by a negative model of the self and a positive model of the other (preoccupied), and one characterized by a positive model of the self and a negative model of the other (dismissing).

Maunder and Hunter [15] suggest that insecure attachment is associated with higher somatic problems because it is associated with ‘increased susceptibility to stress, increased use of external regulators of affect, and altered help-seeking behavior' (p. 556).

In line with this perspective, Meredith and colleagues [16] have proposed the “*Attachment Diathesis Model of Chronic Pain,”* according to which insecure attachment is a specific risk factor for the development of chronic pain; in addition, insecure attachment may influence how the individual deals with the somatic disorder. Specifically, subjects who are preoccupied attached tend to amplify the perception of negative effects as well as the perception of pain.

Moreover, attachment insecurity is also associated with an increased likelihood of depressive symptoms that may in turn impair the possibility to react resiliently to stress, thus, heightening the susceptibility to develop psychosomatic diseases [17, 18].

Research has shown that in the case of FM, there is a higher incidence of depressive symptomatology, a stronger impairment of quality of life, and worst illness perception [19]. Individuals with FM are three times more likely to experience depressive symptoms than the general population [20], with 90% of patients displaying depressive symptoms and 62% receiving a diagnosis of major depression over the course of their illness [21]. Risk for depression may be due, in part, to the deleterious impact of physical symptoms on activities of daily living [22, 23], as chronically ill individuals who perceive a higher impact of disease (i.e., greater perceived negative effect on daily activities and overall QoL) are more likely to exhibit depressive symptoms [24, 25].

### 1.2. Objectives

Although the impact of attachment has been increasingly included in the study of FM, research on the link among attachment insecurities, depression, and QoL is still scarce. In an attempt to further understand the impact of attachment styles on depressive symptoms, and QoL in women with a diagnosis of FM, first, we studied whether the degree of the decrease in QoL of women with FM is related to the severity of depressive symptoms (mild, moderate, and severe). We hypothesized that severe depression will be associated with lower QoL.

Then, we analyzed the differences between FM patients with secure attachment and those with insecure attachment styles (preoccupied, dismissive, and fearful relationship styles). We hypothesized that FM patients with preoccupied and fearful attachment styles would report more depressive symptoms and lower QoL.

## 2. Method

### 2.1. Study Design and Setting

The study was conducted in accordance with the ethical principles contained in the Helsinki Declaration and following the ethical requirements established by National Board of Italian Psychologists Code of Ethics for the Psychologist.

Participants were recruited from online sources. We posted announcements on websites of support care units addressed to women with FM. The announcement specified that we were doing “a research on women with a diagnosis of FM”.

Inclusion criteria were age ≥ 18 years and had previously received a diagnosis of FM.

To participate, the women were required to be at least 18 years old and then offered a linkage to the anonymous online survey. After introducing the goals of the study in detail, the message explained to the women that the survey would take about 10 minutes of their time. If a woman chose to participate and click on the link to the survey, she would be directed to an informed consent document detailing the nature of the survey. After giving consent, the women completed the next two pages of the survey, which included items to evaluate the abovedescribed screening criteria. Women were excluded from the study if they had a diagnosis for other medical conditions and if they were under psychiatric drug.

Women who were eligible were permitted to continue the study. Women who did not meet the eligibility conditions were thanked for their time. All participants were informed of the possibility of interrupting the survey at any time. Women enrolled in the research were given information about data protection and privacy law. The study data was entered into a database (with anonymous codes), to maintain confidentiality, in accordance with local data protection laws and the European Union General Data Protection Regulation (GDPR).

### 2.2. Participants

This cross-sectional exploratory study used a nonprobabilistic convenience sample of 453 Italian women with a physician's diagnosis of FM (average number of years since diagnosis = 6.36; SD = 6.72). The age of the participants ranged from 18 to 75 years, with a mean age of 47 years (SD = 10.91). Most of the participants were married (82%), 6.8% were single, 9.5% were separated, and 1.8% were widowed. Most of women had a high school diploma (75.8%), followed by 17.4% who completed a university degree, and 6.8% a doctorate or postdoctorate degree. Their participations were voluntary, and no incentive was provided.

### 2.3. Measurement

*Attachment styles* were assessed using the Relationship Questionnaire (RQ; [14]; *Italian version*, [26]). The RQ classifies FM patients into one of four different attachment styles: secure, dismissing, preoccupied, and fearful. Participants are required to choose from one of four statements that best describe their predominant attachment style. The RQ as a measure of adult attachment is widely being used by scholars, and it has shown suitable validity as measured with concurrent self- and friend-reports of interpersonal behaviors [14], as well as longitudinal observer-based valuations of behavioral characteristics and personality [27].

*Depression symptoms* were assessed using the Beck Depression Inventory II (BDI-II; [28, 29]; *Italian version*, [30]). Participants' choices are scored from 0 (*absent*) to 3 (*severe*). The total scores range from 0 to 63. Higher scores indicate more severe depression symptoms. The cut-off points for the BDI symptoms are as follows: minimal (0–13), mild (14–19), moderate (20–28), and severe (29–63; [28]). In the current study, the internal consistency coefficient was *α* = 0.84.

*Health-related QoL* was assessed using the Italian version of World Health Organization QoL Questionnaire (WHOQOL-BREF; [31]). It consisted of 26 items of which 24 were distributed in four domains, physical (e.g., routine activities, sleep, and pain), psychological (e.g., self-esteem, religion, and mental status), social relations (e.g., social help and sexual life), and environmental (e.g., financial assets, safety, and information). Two additional items measure overall QoL and overall health. The WHOQOL-BREF has been utilized to evaluate QoL in individuals with chronic fatigue syndrome [12, 32]. Higher scores show a higher perceived QoL. In the current study, the internal consistency coefficient was *α* = 0.83.

### 2.4. Statistical Methods

The statistical analyses were carried out with SPSS software (version 21 for Windows).

Descriptive statistics were computed on the investigated variables, reporting frequencies, percentages, mean values, and standard deviation.

One-way univariate (ANOVA) and multivariate (MANOVA) analysis of variance followed by Tukey's post hoc comparisons were conducted to examine whether BDI-II and WHOQOL-BREF scores differed among the four attachment styles provided by the RQ.

Pearson correlations were used to analyze the association between BDI-II and WHOQOL-BREF scores.

The differences in scores of WHOQOL-BREF in relation to the different severity of depressive symptoms (minimal, mild, moderate, and severe) were examined by MANOVA followed by Tukey's post hoc comparisons.

Effect sizes were estimated using partial eta squared (*η*_p_^2^) [33]. Bonferroni correction for multiple comparisons was applied.

## 3. Results

### 3.1. Descriptive Data

Table 1 shows the frequency, percentages, means, and standard deviations of the analyzed variables.

### 3.2. Main Results

#### 3.2.1. Association between Depressive Symptoms and QoL

Table 2 displays the relationships between BDI-II and WHOQOL-BREF domains and overall QoL/health. High levels of depressive symptoms were associated with significant lower reporting of physical and psychological health, social relations, environmental, and overall QoL/health.  Table 3 presents the correlations among the four domains of WHOQOL-BREF; as observed, there are statistically significant correlations among all domains. There is also a statistically significant correlation between overall QoL/health and the scores obtained from all different domains.

#### 3.2.2. Comparison of QoL according to the Severity of Depressive Symptoms

The one-way MANOVA with depression severity as the independent variable and the four domains and overall QoL/health of WHOQOL-BREF as dependent variables showed a statistically significant main effect *F* (15, 1229) = 7.59; *p* = 0.000, Wilks' *λ* = 0.59, partial *η*^2^ = 0.16 with a large effect.

Given the significance of the overall test, the univariate main effects were examined. Univariate tests showed significant differences between the four severity groups with respect to all four WHOQOL-BREF domains as well as with respect to overall QoL/health with effect sizes (*η*_p_^2^^)^ ranging between 0.21 and 0.40 (Table 3).

Tukey's post hoc test for WHOQOL-BREF domains and total score indicated that those who self-reported a minimal level of depressive symptoms had statistically significantly higher scores on the four domains of WHOQOL-BREF as well as on overall QoL/health compared with the remaining groups. Also, there were statistically significant differences among the mild, moderate, and severe depression groups in all domains of WHOQOL-BREF as well as on overall QoL/health; the mild depression group had significantly lower scores than the moderate and severe groups; and the moderate group had significantly lower scores than the severe group.

#### 3.2.3. Differences in Depressive Symptoms and QoL between Secure and Insecure Attachment Styles

The one-way ANOVA analyses with attachment styles as the independent variable and the BDI-II score as dependent variable showed a significant difference with large effect size (Table 2).

Tukey's post hoc test for BDI-II scores suggested that those who self-reported a secure attachment style had lower levels of depression symptoms compared to dismissive, preoccupied, and fearful ones. No differences were found among the dismissive, preoccupied, and fearful groups on BDI-II score (Table 4).

The one-way MANOVA analysis with attachment styles as the independent variable and the four domains and overall QoL/health of WHOQOL-BREF as dependent variables showed a statistically significant main effect of attachment styles on the dependent variables, *F* (15, 1229) = 5.11; *p* = 0.000, Wilks' *λ* = 0.85, and partial *η*^2^ = 0.05 with small effect.

Given the significance of the overall test, the univariate main effects were examined. Univariate tests showed significant differences between the four attachment styles with respect to all four WHOQOL-BREF domains as well as with respect to overall QoL/health with effect sizes (*η*_p_^2^^)^ ranging between 0.06 and 0.12.

Tukey's post hoc test for WHOQOL-BREF domains and total score indicated that those who self-reported a secure attachment style had higher levels of physical and psychological health, social relations, environmental, and overall QoL/health compared to dismissive, preoccupied, and fearful ones. No differences were among the dismissive, preoccupied, and fearful groups on WHOQOL-BREF domains and overall QoL/health scores (Table 4).

## 4. Discussion

### 4.1. Key Results

The main aim of the present study was to investigate the effect of attachment styles in women with a diagnosis of FM on depressive symptoms and health-related QoL.

It is well demonstrated that both preoccupied and avoidant/dismissing attachment are related with several clinical conditions [34, 35], in particular depression [36–38], that are associated with a low perception of QoL [39].

Also, the pattern of attachment may influence the somatic status since it influences the individuals' response to stress, the intensity of the perception of symptoms, and their ability to seek for social and professional support [40, 41].

### 4.2. Association between Depressive Symptoms and QoL

Our study confirms that depression is associated with chronic pain disorders. In fact, our results showed that the incidence of depressive symptoms in this sample was elevated, with 17% reporting minimal symptoms of depression, 24% reporting mild symptoms of depression, and 59% reporting moderate to severe symptoms of depression.

Wolfe and Michaud [42] have demonstrated the FM patients have higher depression levels compared to individuals suffering from other chronic, musculoskeletal disorders. Certainly, it may be assumed that there are mutual interactions among depression, the perception of pain, and the subjective experience of physical and mental impairment. Therefore, it is crucial to intervene in the presence of depressive symptomatology in order to break the vicious cycle between depression and FM.

### 4.3. Comparison of QoL according to the Severity of Depressive Symptoms

Numerous researches have demonstrated that depression comorbidity negatively influences the prognosis and the QoL of individuals with a diagnosis of FM [9, 20, 22]. It may also be stated that chronic pain, which is one of the main features of FM, can come first and contribute to the onset of depression [43]. Whatever may be the direction of such relationship, our study demonstrated that the impact of severity of depressive symptoms was significant across all domains of QoL: the women with moderate to severe depressive symptoms had the poorest profile regarding all components of QoL.

The consequence of such findings confirms the need to recognize the variety of mechanisms that concur to undermine the person's QoL in order to plan targeted and efficacious interventions.

### 4.4. Differences in Depressive Symptoms and QoL between Secure and Insecure Attachment Styles

Our study suggests that insecure attachment styles may be risk factors for depressive symptoms in women with FM. Specifically, women with dismissive, preoccupied, and fearful attachment styles had significantly higher depressive symptoms compared to women with a secure attachment style.

In particular, the BDI-II mean score for women with dismissive, preoccupied, and fearful attachments exceeded the clinical threshold for depression (moderate depressive symptoms), whereas securely attached women had depression scores in the “mild depression” range as assessed through the BDI-II.

Specifically, we may hypothesize that people with preoccupied and fearful patterns, who view themselves as fragile and the environment as threatening, may perceive higher levels of stress and more intense, severe symptoms [44]. On the other hand, dismissing attached individuals, characterized by low trust in the others and poor emotional involvement, may delay the access to treatment, minimizing the impact of their symptoms [18].

Several studies showed that individuals with FM have decreased or compromised QoL in comparison to healthy age-matched controls [45].

Consistent with the attachment literature [12, 18] and our hypotheses, our study results suggest that insecure attachment styles may be an additional risk factors for QoL of women with FM. Specifically, women with dismissive, preoccupied, and fearful attachment styles had significantly lower levels of QoL compared to women with a secure attachment style.

We may suppose that FM patients with a negative representation of themselves and who hyperreact to stresses, on the one hand, and FM patients who are rigidly self-reliant and tend to ignore negative feelings, on the other hand, are more likely to experience poor wellness in terms of physical and psychological health, personal adjustment, and social relationships [46].

### 4.5. Limitations

Despite the interesting results regarding the associations among attachment styles, QoL, and depressive symptoms in women with FM, these findings should be interpreted with caution due to the following limitations.

First, the cross-sectional design of this research does not allow any stable conclusions and precludes any inference of causality. The outcomes should be verified and supported by further investigations, including longitudinal research.

Second, all measures were based on self-reports, so we could not prevent social desirability bias. The accuracy of self-reported attachment styles may be improved by using interview-based measure of attachment; for example, AAI (the Adult Attachment Interview) [47].

### 4.6. Interpretation

Our study demonstrates that, when evaluating the impact of FM on the QoL of women, it is important to consider the complexity of the variables that are at play. In particular, insecure attachment styles and depressive symptoms seem to increase the likelihood of the psycho-social-somatic malaise in FM patients. For such reasons, it is important to include measures of attachment style and depression symptoms to identify women at higher risk for their health and functioning.

Clinicians may assist their FM patients in recognizing how their attachment patterns may be influencing their capacity to regulate their effects, manage negative emotions, and compromise the use of adaptive strategies to face physical and psychic stressors. Interventions focusing on attachment may provide a useful tool to enhance the patient's capacity to respond resiliently to the complex challenges of a multidetermined disorder such as FM.

## Figures and Tables

**Table 1:** Descriptive statistics (*n* = 453).

Attachment styles (RQ)	
Secure, *N* (%)	121 (26.7%)
Dismissing, *N* (%)	88 (25.6%)
Preoccupied, *N* (%)	116 (25.6%)
Fearful, *N* (%)	128 (28.3%)
Depression symptoms (BDI-II)	
Mean score (SD)	22.56 (9.40)
Minimal (0–13), *N* (%)	79 (17.4%)
Mild (14–19), *N* (%)	109 (24.1%)
Moderate (20–28), *N* (%)	155 (34.2%)
Severe (29–63), *N* (%)	110 (24.3%)
Quality of life (WHOQOL-BREF)	
Physical health–mean score (SD)	2.88 (.50)
Psychological health–mean score (SD)	2.54 (.68)
Social relationships–mean score (SD)	2.51 (.73)
Environment–mean score (SD)	2.50 (.55)
Overall QoL/health–mean score (SD)	2.60 (.48)

**Table 2:** Correlation coefficients among BDI-II, four domains of WHOQOL-BREF, and overall QoL/health.

	1	2	3	4	5	6
1.BDI-II scores	_					
2. WHOQOL-BREF physical health	-0.522^∗^	_				
3. WHOQOL-BREF psychological health	-0.569^∗^	0.506^∗^	_			
4. WHOQOL-BREF social relationships	-0.498^∗^	0.455^∗^	0.549^∗^	_		
5. WHOQOL-BREF environment	-0.539^∗^	0.485^∗^	0.567^∗^	0.523^∗^	_	
6. WHOQOL-BREF overall QoL/health	-0.669^∗^	0.763^∗^	0.819^∗^	0.742^∗^	0.847^∗^	_

^∗^ *p* < 0.001.

**Table 3:** Comparison of WHOQOL-BREF mean scores according to the severity of depressive symptoms.

	Minimal	Mild	Moderate	Severe		
	Mean score (SD)	Mean score (SD)	Mean score (SD)	Mean score (SD)	*F*	*η* ^2^
WHOQOL-BREF physical health	3.22 (0.47)^bcd^	3.03(0.45)^acd^	2.87 (0.41)^abd^	2.51 (0.45)^abc^	47.60^∗^	0.24
WHOQOL-BREF psychological health	3.15 (0.62)^bcd^	2.72 (0.63)^acd^	2.46 (0.50)^abd^	2.04 (0.60)^abc^	61.02^∗^	0.29
WHOQOL-BREF social relationships	3.06 (0.68)^bcd^	2.71 (0.70)^acd^	2.41 (0.62)^abd^	2.08 (0.64)^abc^	38.81^∗^	0.21
WHOQOL-BREF environment	3.00 (0.52)^bcd^	2.63 (0.51)^acd^	2.44 (0.42)^abd^	2.11 (0.45)^abc^	59.14^∗^	0.28
WHOQOL-BREF overall QoL/health	3.09 (0.43)^bcd^	2.76 (0.41)^acd^	2.54 (0.30)^abd^	2.18 (0.47)^abc^	101.30^∗^	0.40

^∗^ *p* < 0.001.

**Table 4:** Univariate and post hoc analyses comparing the mean and standard deviation (SD) for attachment style on BDI-II and WHOQOL-BREF scores.

	Secure	Dismissing	Preoccupied	Fearful		
	Mean score (SD)	Mean score (SD)	Mean score (SD)	Mean score (SD)	*F*	*η* ^2^
BDI-II scores	17.55 (8.08)^bcd^	23.82 (9.85)^a^	26.84 (8.90)^a^	23.58 (9.49)^a^	21.27^∗^	0.13
WHOQOL-BREF physical health	3.10 (0.47)^bcd^	2.81(0.56)^a^	2.76 (0.45)^a^	2.83 (0.44)^a^	11.66^∗^	0.072
WHOQOL-BREF psychological health	2.82 (0.68)^bcd^	2.42 (0.62)^a^	2.41 (0.69)^a^	2.48 (0.66)^a^	9.64^∗^	0.060
WHOQOL-BREF social relationships	2.82 (0.67)^bcd^	2.45 (0.78)^a^	2.36(0.69)^a^	2.51 (0.73)^a^	10.17^∗^	0.064
WHOQOL-BREF environment	2.81 (0.52)^bcd^	2.42 (0.56)^a^	2.32 (0.46)^a^	2.50 (0.52)^a^	20.70^∗^	0.121
WHOQOL-BREF overall QoL/health	2.88 (0.46)^bcd^	2.51 (0.49)^a^	2.46 (0.41)^a^	2.52 (0.41)^a^	21.18^∗^	0.124

^∗^ *p* < 0.001. Note: superscripts refer to significant comparisons (^a^secure, ^b^dismissing, ^c^preoccupied, ^d^fearful).

## Data Availability

The datasets presented in this article are not readily available because our data are identifiable. Requests to access the datasets should be directed to cristina.sechi@unica.it.
